# A novel genetically-obese rat model with elevated 11beta-hydroxysteroid dehydrogenase type 1 activity in subcutaneous adipose tissue

**DOI:** 10.1186/1476-511X-9-132

**Published:** 2010-11-17

**Authors:** Sakamuri SS Vara Prasad, Anamthathmakula Prashanth, Chodavarapu Pavan Kumar, Sirisha J Reddy, Nappan V Giridharan, Ayyalasomayajula Vajreswari

**Affiliations:** 1 Department of Biochemistry, National Institute of Nutrition, Jamai Osmania PO, Hyderabad-500 604, Andhra Pradesh, India; 2 National center for Laboratory Animal Sciences, Indian Council of Medical Research, Jamai Osmania PO, Hyderabad-500 604, Andhra Pradesh, India

## Abstract

11β-hydroxysteroid dehydrogenase type 1 (11β-HSD1) catalyzes the conversion of inactive glucocorticoids to active glucocorticoids and plays an important role in the development of obesity and metabolic syndrome. 11β-HSD1 activity is lower in liver and higher in omental adipose tissue of obese rodent models like obese zucker rats, *Ob/Ob *and *db/db *mice. Here, we report the 11β-HSD1 activity in liver and adipose tissue of lean and obese rats of WNIN/Ob strain, a new genetic rat model of obesity. 11β-HSD1 activity in liver, omental and subcutaneous adipose tissues of 3 month-old male WNIN/Ob lean and obese rats was assayed. As observed in other rodent models, 11β-HSD1 activity was lower in liver and higher in omental adipose tissue. In contrast to other rodent obese models, WNIN/Ob obese rats had elevated 11β-HSD1 activity in subcutaneous adipose tissue, which is in line with the observation in human obesity. Here, we conclude that dysregulation of 11β-HSD1 in WNIN/Ob obese rat model is identical to human obesity, which makes it an excellent model for studying the effect of 11β-HSD1 inhibitors in ameliorating obesity and metabolic syndrome.

## Introduction

Glucocorticoids are essential for the regulation of metabolism, normal functioning of nervous, cardiovascular, skeletal and immune systems. They are also implicated in the pathogenesis of obesity and metabolic syndrome as evidenced by the fact that, elevated systemic glucocorticoid concentration in Cushing's syndrome results in the development of metabolic syndrome including visceral obesity. The role of plasma glucocorticoids in the development of idiopathic obesity is not clear, as their levels are not altered or sometimes even low in obese patients [1].

Tissue sensitivity to glucocorticoids depends on plasma hormone levels, density of glucocorticoid receptors and local metabolism of glucocorticoids by 11β-hydroxysteroid dehydrogenases. 11β-hydroxysteroid dehydrogenase type 1 (11β-HSD1) catalyzes the conversion of inactive glucocorticoids (cortisone in humans and 11-dehydrocorticosterone in rodents) to active glucocorticoids (cortisol in humans and corticosterone in rodents) [2]. It is highly expressed in liver, adipose tissue and brain [2]. Another enzyme, 11β-HSD2 catalyses the reverse reaction and expressed in distal nephron, sweat and salivary glands [3].

Recently, it has been reported that 11β-HSD1 plays an important role in the development of obesity and insulin resistance. In obese zuckar rats and in obese human subjects, 11β-HSD1 activity is higher in adipose tissue, where as in liver it is low [4-6]. The role of 11β-HSD1 in obesity and metabolic syndrome is further supported by the transgenic animal model studies. Adipose-specific overexpression of 11β-HSD1 in mice resulted in the development of majority of metabolic syndrome features including visceral obesity and insulin resistance [7], where as 11β-HSD1 knock-out in mice resulted in the development of resistance to diet-induced obesity [8]. Recently, selective inhibitors of 11β-HSD1 are developed to treat obesity and metabolic syndrome.

Rodent models of obesity provide valuable information on the molecular mechanisms underlying the development of obesity and its associated complications. WNIN/Ob rat obese strain is developed from 80-year-old inbred wistar rat colony at National centre for Laboratory Animal Sciences (Hyderabad, India) [9]. They exhibited all biochemical characteristics of leptin-resistant obese zuckar rats. These rats are hyperphagic, hyperinsulinemic, hyperleptinemic and have dyslipidemia [9]. Preliminary studies on WNIN/Ob obese rats show no molecular defects in leptin and leptin receptor [unpublished data]. Further, molecular mutation leading to the development of obesity in these obese rats is under investigation.

Here, we hypothesize that WNIN/Ob obese rats have altered 11β-HSD1 enzyme activity in liver and adipose tissue as observed in other obese rodent models. To test our hypothesis, we studied the 11β-HSD1 enzyme activity in liver, adipose tissue and skeletal muscle of 3 month-old WNIN/Ob lean and obese rats.

## Materials and methods

### Animals

3 month-old WNIN/Ob male lean and obese rats (n = 6) were obtained from National Centre for Laboratory Animal Sciences (NCLAS, India) and study was approved by Institutional Animal Ethical Committee. Animals were acclimatized in individual cages under controlled conditions of temperature (22 ± 1°C), humidity (50-60%) and light (12:12 h light-dark cycle). Stock diet and water were provided *ad libitum*. After overnight fasting, blood was collected and the animals were sacrificed by decapitation. Tissues were dissected out, weighed and stored at -80°C until the analysis.

### Adiposity Index (%)

Adiposity index was calculated by dividing total weight of omental, retroperitoneal and epididymal adipose tissue with the total bodyweight and multiplied by 100.

### Plasma parameters

Plasma corticosterone (Siemens, Los Angeles, USA), insulin (BARC, India) and leptin (Linco Research, USA) levels were measured by radioimmunoassay (RIA). Plasma glucose and triglyceride levels were measured by Commercial Kits (Biosystems, Spain). Tumor necrosis factor-α (TNF- α), Interleukin-6 (IL-6) and Macrophage chemoattractant protein-1 (MCP-1) were estimated by Milliplex Rat cytokine immunoassay kit (Millipore, USA).

### Insulin resistance and glucose tolerance

Insulin resistance was assessed from homeostasis model assessment of insulin resistance (HOMA-IR). HOMA-IR was calculated from fasting glucose and insulin values using the following formula.

HOMA−IR=(fasting insulin[μU/ml]× fasting glucose [mmol/l])/22.5

Oral glucose tolerance test was performed to measure the glucose tolerance. After an overnight fast, glucose (200 g/l) was administered oro-gastrically at a dose of 2.0 g/kg body weight and blood samples were collected from supraorbital at 0, 30, 60 and 120 min. Glucose and insulin levels were measured at all time points.

Glucose tolerance was calculated during OGTT by calculating Area under curve (AUC) for insulin and glucose by the trapezoidal method [10].

### 11β-HSD1 activity

11β-HSD1 functions as a reductase in vivo, reactivating corticosterone from inactive 11-dehydrocorticosterone. However, in tissue homogenates, dehydrogenase activity predominates, hence 11β-HSD1 activity was measured by conversion of corticosterone to 11-dehydrocorticosterone [11]. Post nuclear fractions from liver and omental adipose tissue were prepared by centrifuging tissue homogenate at 1000 g for 20 min. 11β-HSD1 activity was measured in post nuclear fractions of liver and omental adipose tissue by incubating in duplicates at 37° C, in Krebs-Ringer buffer containing 0.2% glucose, 1 mM NADP and 50 nM 1, 2, 6, 7-[^3^H_4_] corticosterone (Amersham, UK). Conditions were optimized to ensure first order kinetics, by adjusting protein concentrations for liver (40 μg/ml), adipose tissue (1 mg/ml) and skeletal muscle (1 mg/ml). After incubation (30 min for liver and 6 h for adipose tissue and skeletal muscle), steroids were extracted with ethyl acetate. Ethyl acetate was evaporated under dry nitrogen and steroids were resuspended in mobile phase (50% water, 30% acetonitrile and 20% methanol). Steroids were separated by HPLC using reverse phase C18 column and radioactive counts from substrate and product peaks were calculated by online scintillation counter (IN/US systems, UK). Enzyme activity was expressed as percentage of substrate conversion.

### Statistical analysis

Results were expressed as means±S.E of six animals from each phenotype. Statistical significance was determined by student's t-test and comparisons were made between lean and obese phenotype.

## Results

### Body weights, tissue weights and adiposity index

At 3-months of age, WNIN/Ob obese rats had significantly higher bodyweights (1.7 fold) as compared to their lean counter parts (Table 1). Adiposity index was significantly higher in obese rats (5.4 fold) as compared to their age and sex-matched lean rats (Table 1). Weights of liver, retroperitoneal, epididymal and omental fat depots were also significantly higher (1.7 fold, 5.4 fold, 7.3 fold and 8.6 fold respectively) in obese rats as compared to lean rats (Table 1). Adrenal weights were significantly higher (1.3 fold) in obese rats as compared to lean rats, where as adrenal to bodyweight ratio were significantly lower (0.7 fold) in obese rats as compared to lean rats (Table 1).

**Table 1:** Physical parameters in 3 month-old WNIN/Ob lean and obese rats.

	Lean (n = 6)	Obese (n = 6)
Body wt (g)	307 ± 21	543 ± 13*
Adrenal wt (mg)	37.8 ± 1.9	48.0 ± 1.5*
Adrenal/body wt	0.12 ± 0.004	0.09 ± 0.004*
Adiposity index (%)	1.0 ± 0.03	5.60 ± 0.04*
Liver (g)	10.3 ± 0.37	17.6 ± 0.04*
Omental adipose tissue (g)	0.36 ± 0.04	1.96 ± 0.06*
Epididymal adipose tissue (g)	1.44 ± 0.12	10.5 ± 0.08*
Retroperitoneal adipose tissue (g)	1.84 ± 0.24	15.8 ± 0.04*

Values are mean± S.E of 6 rats. Values with * mark are significant at P < 0.05 level (by student's t test). Comparisons were made between lean and obese phenotypes.

### Plasma parameters

Plasma corticosterone levels were significantly elevated (2 fold) in obese rats as compared to those of lean rats (Table 2). Fasting insulin and leptin levels were also significantly higher (29.4 and 14.2 fold respectively) in obese rats compared to lean rats (Table 2). Plasma triglyceride levels were significantly higher (4.5 fold) in obese rats as compared to lean rats (Table 2). There were no significant changes in the plasma levels of MCP-1 and IL-6 in obese rats as compared to their lean counter parts (Table 2). Although TNF-α level was detectable in lean rats, it was not in detectable range in obese rats (Table 2).

**Table 2:** Plasma parameters in 3 month-old WNIN/Ob lean and obese rats.

	Lean (n = 6)	Obese (n = 6)
Corticosterone (ng/ml)	213 ± 34	433 ± 50*
Insulin (μU/ml)	7.0 ± 3.6	206 ± 23*
Triglycerides (mg/dl)	46.5 ± 3.0	212 ± 29*
Glucose (mg/dl)	80.0 ± 2.0	86.0 ± 4.7
HOMA-IR	1.4 ± 0.8	52.5 ± 7.0*
Glucose AUC (mmol/l)	304 ± 13	472 ± 31*
Insulin AUC (μU/ml)	5.1 ± 1.2	32 ± 2.8*
Glucose AUC/Insulin AUC (× 1000)	74 ± 14	15 ± 1.4*
Leptin (ng/ml)	1.0 ± 0.1	14.2 ± 1.8*
TNF-α (pg/ml)	3.0 ± 1.6	Nd
IL-6 (ng/ml)	191 ± 62	57.5 ± 35
MCP-1 (μg/ml)	0.3 ± 0.1	2.5 ± 2.3

Values are mean± S.E of 6 rats. Values with * mark are significant at P < 0.05 level (by student's t test). Comparisons were made between lean and obese phenotypes. Nd, non-detectable.

### Insulin resistance and glucose tolerance

Insulin resistance calculated by HOMA-IR is significantly higher (37 fold) in obese rats compared to age and sex-matched lean rats (Table 2). Glucose AUC and insulin AUC were significantly higher (1.5 fold and 6.2 fold respectively) in obese rats compared to lean rats (Table 2). Glucose to insulin AUC ratio was significantly lower (4.9 fold) in obese rats compared to lean rats (Table 2).

### 11β-HSD1 activity in omental and subcutaneous adipose tissue

**Figure 1:**
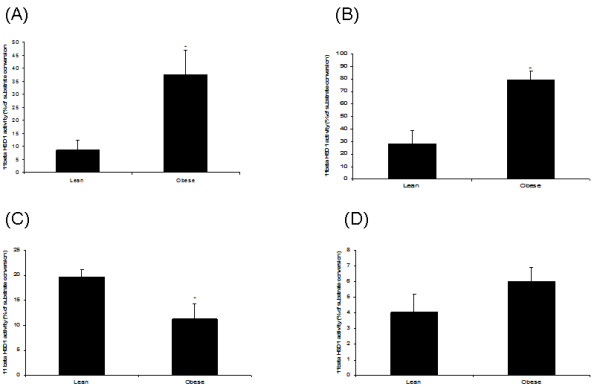
**11β-HSD 1 activity in 3 month-old male WNIN/Ob lean and obese rats**. (A). Omental fat. (B). Subcutaneous fat. (C). Liver. (D). Quadriceps muscle. Values are means ± S.E for 6 rats. Mean values with * mark are significant at P < 0.05 level (by student's t test). Comparisons were made between lean and obese phenotypes.

11β-HSD1 activity was significantly higher (4.4 fold, 2.8 fold respectively) in omental and subcutaneous adipose tissues of 3-month old WNIN/Ob obese rats as compared to those of age and sex-matched lean rats (Figure 1A&1B).

### 11β-HSD1 activity in liver and skeletal muscle

11β-HSD1 activity was significantly lower (0.6 fold) in liver of obese rats as compared to that of age and sex-matched lean rats (Figure 1C). Skeletal muscle (quadriceps) 11β-HSD1 activity was not different between 3 month old WNIN/Ob lean and obese rats (Figure 1D).

## Discussion

In this study, we reported 11β-HSD1 enzyme activity in the peripheral tissues of novel genetically-obese rats of WNIN/Ob strain. WNIN/Ob obese rats had higher plasma corticosterone levels and heavier adrenal glands, indicating elevated hypothalamus-pituitary-adrenal (HPA) axis activity. They also have lower11β-HSD1 activity in liver and higher activity in omental and subcutaneous adipose tissue. To our knowledge, this is the first genetic rodent model of obesity, exhibiting higher 11β-HSD1 activity in subcutaneous adipose tissue.

Glucocorticoids play an important role in adipocyte biology. Active glucocorticoids, cortisol (in humans) and corticosterone (in rodents) are essential for the differentiation of pre-adipocytes into mature adipocytes [12]. 11β-HSD1 in adipose tissue is known to increase the local concentration of active glucocorticoids and thereby induce preadipocyte differentiation. This is further supported by cell culture studies, where 11β-HSD1 inhibition has been shown to decrease cortisone-induced differentiation of pre-adipocytes [13]. Further, the role of 11β-HSD1 in adipocyte differentiation and in obesity is confirmed by transgenic studies. Transgenic mice over-expressing 11β-HSD1 in adipose tissue have developed visceral obesity with increased preadipocyte differentiation [7]. In leptin-resistant obese zucker rats, 11β-HSD1 activity is elevated in omental adipose tissue and no change is observed in subcutaneous adipose tissue [4]. In leptin-deficient *ob/ob mice *and in leptin-resistant *db/db *mice, 11β-HSD1 activity is higher in omental adipose tissue and lower in subcutaneous adipose tissue [10]. In recently reported polygenic obese mice model and in diet-induced obese mice, 11β-HSD1 activity is lower in omental and subcutaneous adipose tissue [14,15]. In human obesity, 11β-HSD1 activity is higher in subcutaneous adipose tissue, however in omental adipose tissue the observations are inconsistent [5,6]. The newly studied WNIN/Ob obese rat had elevated 11β-HSD1 activity in omental adipose tissue. They also had higher enzyme activity in subcutaneous tissue, which is contrast to the observations made with other obese rodent models, and in agreement with the observations in human obesity. Although these obese rats had higher enzyme activity in omental and subcutaneous adipose tissue under fasting condition, interestingly, the enzyme activity is decreased significantly in fed-state as compared to lean rats (unpublished data).

In addition to the gene mutation that caused obesity, the observed higher 11β-HSD1 activity in omental and subcutaneous adipose tissue, might contribute to the development of obesity in this model. The exact mechanism responsible for the observed higher 11β-HSD1 activity in adipose tissue of WNIN/Ob obese rats can not be explained, unless the mutation is identified. However, the observed higher plasma corticosterone, insulin and leptin levels might be responsible for enhanced 11β-HSD1 activity, as these molecules upregulate 11β-HSD1 expression in adipocytes [16-18].

Glucocorticoids antagonize insulin signaling in various tissues and thus implicated in the development of insulin resistance [19]. 11β-HSD1 negatively influences tissue insulin sensitivity by generating local active glucocorticoids. 11β-HSD1 knockout mice have improved insulin sensitivity, on the contrary transgenic overexpression of 11β-HSD1 in adipose tissue of mice results in insulin resistance [7,20]. In obese zuckar rats, increased 11β-HSD1 activity in adipose tissue is associated with elevated fasting plasma insulin level [4]. Similar to obese zucker rat, WNIN/Ob obese rats have increased peripheral insulin resistance as indicated by elevated fasting insulin, HOMA-IR and increased glucose AUC. Despite of decreased peripheral insulin sensitivity, WNIN/Ob obese rats are not glucose intolerant, as glucose level reaches to normal at the end of second hour after oral glucose challenge (data not shown). As the obese rats have elevated 11β-HSD1 activity in adipose tissue, increased active glucocorticoids in adipose tissue might be responsible for the observed insulin resistance in this model.

Adipose tissue secrets various signaling molecules including cytokines like TNF-α and IL-6 [21]. Previous studies reported elevated levels of plasma TNF- α and IL-6 in obese rodent models and in humans, which are implicated in the development of obesity induced insulin resistance and diabetes [22,23]. Transgenic mice over-expressing 11β-HSD1 in adipose tissue has elevated TNF- α mRNA and plasma TNF- α, where as 11β-HSD1 knock-out mice have decreased TNF- α mRNA in adipose tissue [24]. Recently, knock-down of 11β-HSD1 in pre-adipocytes has resulted in the decreased expression of IL-6 [25]. Although WNIN/Ob obese rats have elevated 11β-HSD1 activity in adipose tissue, plasma levels of TNF-α and IL-6 levels are not elevated. This contrasting observation could be due to activation of other signaling mechanisms that can downregulate expression of TNF- α and IL-6 in adipose and other tissues. Secretion of MCP-1 by adipose tissue is the key step in the recruitment of macrophages. In obesity, elevated plasma and adipose MCP-1 levels are reported along with increased tissue macrophages [26]. 11β-HSD1 inhibition has been shown to decrease MCP-1 expression in adipose tissue [27]. Although plasma MCP-1 levels showed a trend towards increase, the observation is not statistically significant due to large variation.

Liver exhibits significant 11β-HSD1 activity and is also the main organ for the conversion of inactive glucocorticoids to active glucocorticoids [28]. Functional studies have shown that transgenic overexpression of 11β-HSD1 in liver results in insulin resistance, hepatic steatosis without causing obesity [29]. On the contrary, 11β-HSD1 knock-out mice are resistant to high fat diet-induced metabolic syndrome [20]. Previous studies involving rodent models of obesity and in obese humans have reported lower 11β-HSD1 activity in liver, which is considered to be a compensatory mechanism to improve obesity associated insulin resistance [4-6]. In the present study, WNIN/Ob obese rats displayed lower hepatic 11β-HSD1 activity, which is in line with the previous observations.

Recently, we have also studied the impact of nutrients like vitamin A and polyunsaturated fattyacids on 11β-HSD1 activity in WNIN/Ob obese rats. Chronic challenging with vitamin A enriched diet, decreased 11β-HSD1 activity in liver and adipose tissue of obese rats, with concomitant reduction in adiposity (unpublished data). A decreased trend in 11β-HSD1 activity in adipose tissue of obese rats was also observed with chronic feeding of diet-rich in n-6 polyunsaturated fattyacids (unpublished data).

In conclusion, WNIN/Ob obese rats have elevated levels of circulatory glucocorticoids, lower hepatic 11β-HSD1 activity and higher omental and subcutaneous HSD1 activity. WNIN/Ob obese rats show several biochemical and physiological features similar to human obesity, particularly with regard to tissue glucocorticoid metabolism. Based on these observations, we propose that, WNIN/Ob obese rat not only serve as a good model to study the role of 11β-HSD1 in the development of obesity and metabolic syndrome, but also to study the effect of 11β-HSD1 inhibitors and 11β-HSD1 regulating nutrients in the amelioration of obesity and metabolic syndrome.

## Abbreviations

11β-HSD1: 11β-hydroxysteroid dehydrogenase type 1.

## Competing interests

The authors declare that they have no competing interests.

## Authors' contributions and information

SSSVP involved in the animal handling, tissue dissection, plasma assays, and enzyme assay and prepared the first draft. AP and JSR involved in cytokine assays, PKC involved in the enzyme assay. GNV involved in the animal breeding and provided WNIN/Ob obese rats. VA drafted the manuscript and had overall supervision and gave final approval of the manuscript to be published. All authors have read and approved the final manuscript.
